# The influence of pregnancy classes on the use of maternal health services in Indonesia

**DOI:** 10.1186/s12889-020-08492-0

**Published:** 2020-03-20

**Authors:** Khadijah Azhar, Ika Dharmayanti, Dwi Hapsari Tjandrarini, Puti Sari Hidayangsih

**Affiliations:** Center for Research of Public Health Effort, National Institute of Health Research and Development, Ministry of Health, Jalan Percetakan Negara 23, Jakarta, 29 Indonesia

**Keywords:** Pregnancy class, Antenatal care, Maternal health, Skilled birth attendant, Facility-based delivery

## Abstract

**Background:**

Indonesia has developed the pregnancy class program for mothers in an effort to reduce the high maternal mortality rate. This study aims to understand the influence of pregnancy classes on mothers’ use of maternal and neonatal health services, which are known to improve pregnancy and delivery outcomes.

**Methods:**

This study used data on members of households in communities in Indonesia, based on the 2016 National Health Indicators Survey (Sirkesnas), which covered 34 provinces and 264 districts/cities. The analysis focused on a sample of women ages 10–54 years who had ever been married and had given birth in the previous 3 years. The study analyzed three behaviors as outcome variables: whether a mother had adequate antenatal care, used a skilled birth attendant, and had a facility-based delivery. Logistic and multinomial logistic regression analysis was used to explore those relationships.

**Results:**

29% of mothers utilized adequate antenatal care (a minimum of five antenatal care components and at least four antenatal care visits), 77% of mothers used skilled birth attendants for delivering their baby, and 76% of mothers used a health facility to give birth. Only 7% of mothers participated in the complete pregnancy class program. Mothers who completed participation in the pregnancy class program had 2.2 times higher odds of receiving adequate antenatal care [OR = 2.19; 95% CI: 1.62 to 2.97; *P* < 0.001]. Those who completed participation in the class had 2.7 times higher odds of using skilled birth attendants for delivery [OR = 2.69; 95% CI: 1.52 to 4.76; *P* < 0.001] and 2.8 times higher odds of giving birth in a health facility compared to a non-health facility [OR = 2.77; 95% CI: 1.56 to 4.91; *P* < 0.001].

**Conclusions:**

Participation in pregnancy classes was positively associated with utilization of adequate antenatal care, skilled birth attendants, and delivery at health facility. Since participation in pregnancy classes in positively associated with maternal healthcare utilization, policy efforts should focus on improving implementation of the KIH program at the local level.

## Background

Maternal mortality rates are still quite high in most developing countries and occur at a higher rate among poor mothers and among those who reside in rural areas [1]. Women die as a result of complications during and following pregnancy and childbirth [1]. To avoid maternal deaths, it is important to prevent unwanted pregnancies and ensure that all births are attended by skilled health professionals, and that all women have adequate access to maternal health services [1]. In addition, as highlighted in Thaddeus and Maine’s framework, pregnant women face three delays in receiving adequate care that might cause maternal death: (1) the decision to seek care; (2) the ability to reach a health facility; and (3) the provision of adequate care at the facility [2].

Indonesia’s overall maternal mortality rate (MMR) remains high as of 2015, at an estimated 305 deaths per 100,000 live births [3] —far from the Millennium Development Goals target of 102 death per 100,000 live births [4]. Furthermore, reaching the Sustainable Development Goals (SDGs) target of 70 deaths per 100,000 live births in 2030 [5] would require extensive effort from Government of Indonesia (GOI). One of the causes of the high MMR in Indonesia is low maternal knowledge about healthcare and low ability to recognize obstetric danger signs, which may hinder a mother’s decision to seek care [6]. To improve education on maternal and neonatal health (MNH), the GOI began implementing a health promotion program in 2009—the pregnancy class program (*Kelas Ibu Hamil*, or KIH) [7]. The KIH program aims to improve pregnant women’s understanding of pregnancy, their use of antenatal care (ANC) services and postpartum family planning, as well as improve awareness of infectious diseases [8–12]. The target population for KIH is women who are 22–36 weeks pregnant. Each class includes a maximum of 10 pregnant women and is facilitated by midwives or health workers who have received appropriate MNH training [13].

**Fig. 1:**
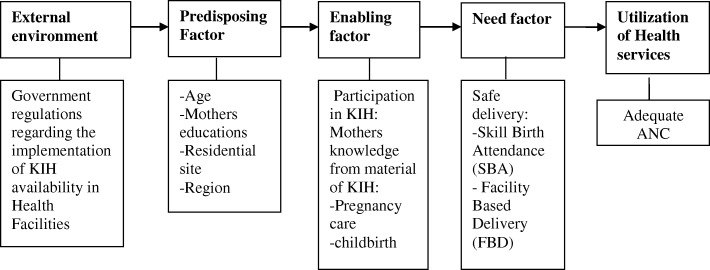
Theoritical framework of factors associated with KIH and the utilization of ANC, SBA and FBD

Figure 1 illustrates the modified Andersen behavioral theoretical framework of factors associated with KIH and the utilization of ANC, skilled birth attendance (SBA) and facility-based delivery (FBD) [14]. KIH is one mechanism available for mothers to get various important information related to pregnancy and birth. Appropriate knowledge about pregnancy and childbirth may influence mothers to use adequate ANC. Mothers who have participated in KIH prefer delivery with the aid of SBA and prefer to deliver in the facility. In relation with Thaddeus and Maine’s framework [14], KIH may increase mothers’ knowledge on pregnancy and may reduce the time taken to recognize danger signs and make decisions to seek care (delay 1).

Few studies have been conducted to evaluate the impact of the KIH program in improving pregnant women’s knowledge, and those studies have been both small in scale and limited to one district [15–18]. One qualitative study that examined KIH implementation in Batang District found that the following increased after attending KIH programs: pregnant women’s knowledge, attitudes, and actions regarding delivery preparedness; postpartum and neonatal care; and rates of choosing contraception [17]. Other evidence in Tegal, Central Java, found that KIH participation was associated with a decreased risk of pregnancy complications; however, this finding was not statistically significant [11].

Although the results generally suggest that KIH improves pregnant women’s knowledge, the consensus is that implementation of the program is still weak [16, 17, 19, 20]. Evidence shows that factors affecting program implementation include a lack of high-quality health services at the facility level such as lack of midwives trained for the KIH program, insufficient infrastructure such as space or room, absence of monitoring and evaluation of the program, and a lack of information dissemination regarding the pregnancy classes to pregnant women in the community [17]. Those studies show that most pregnancy classes lack coordination, have limited family participation, and are implemented with unclear schedules [16–20].

Given the large evidence gap surrounding the influence of the KIH program on care-seeking behaviour at the national level, this study aims to understand the influence of pregnancy classes on mothers’ use of MNH services along the continuum of care, including ANC, delivery in a health facility, and use of SBAs using the 2016 National Health Indicators Survey. We assess whether the KIH program significantly influences mothers in obtaining a complete package of MNH services. We hypothesize that mothers who participated completely in the KIH program would more often use ANC, health workers as birth attendants and deliver at health facilities—in other words, that the KIH program would help prevent the first delay in the Thaddeus and Maine’s framework.

## Methods

### Design, sample, and setting

This study used the 2016 National Health Indicators Survey (Sirkesnas) as its main source of data. The Sirkesnas is a nationally representative survey conducted by the National Institute of Health Research and Development, Indonesian Ministry of Health; it covers 34 provinces and 264 districts/cities and collects quantitative information at three levels of observation: individual, household, and health facility, including at the district health office. Overall, Sirkesnas 2016 assesses nine main health areas, one of which is maternal health [21].

The selection of Sirkesnas respondents was based on 2010 Population Census blocks. The study used simple random sampling to select 25 households in 1200 census blocks, totalling 30,000 households. Households that had children aged less than 5 years were prioritized in the sampling. In total, Sirkesnas 2016 collected information on 97,986 individuals and 22,795 households [21].

The information used in this study was retrieved from individual questionnaires, specifically Section E—Mother’s Health Services. This section was designed specifically for women ages 10–54 years who reported ever having been married and had given birth in the 3 years before the survey. This section only collected the information of mothers’ last birth. In total, there were 6790 mothers who answered this section, comprising our total sample.

### Measures

#### Maternal health service variables

We used three variables to assess the influence of the KIH program in promoting care-seeking behaviour that leads to safe pregnancy, delivery, and postpartum management. The three behaviors analysed were whether a mother utilized adequate ANC, used an SBA, and had a facility-based delivery. The details of each variable are as follows:

1. Adequate number of ANC visits and types of examination, hereinafter referred to as adequate ANC, defined as at least four visits during pregnancy (at least once in the first and second trimesters, and twice in the last trimester) and receipt of at least five type of health services or examinations.
2. SBA utilization for the last contact, defined as the use of a health professional (midwife or doctor, either at home or in a health facility) for the mother’s last contact in delivery. The culture surrounding use of traditional birth assistance (TBA) is still strong in Indonesia and government of Indonesia is reducing their involvement by integrating TBAs in a mother’s birth procedure. TBAs are allowed to accompany mothers in the delivery but not to directly assist. Usually, a mother will first use TBAs, who will then refer them to an SBA such as a midwife or directly to the health facility. The Sirkesnas was designed to capture this condition by assigning the first person who helps a mother during her delivery as the first contact and assigning the last person who assists the mother in her delivery as the last contact. It is highly possible that the first and last contact SBA are the same person. In situations in which a mother’s first contact was with a non-health worker, however she was accompanied by a health worker, we classified the mother as using SBA for her last contact.
3. FBD utilization, defined as the use of a healthcare facility for delivery in the following three categories—health center, hospital, and non-health facility (home and prior arriving the health facilities delivery).

#### Explanatory variables and covariates

The explanatory variable in this study was the degree of a women’s participation level in the KIH program, assessed as three categorical decisions: no participation, incomplete participation, and complete participation. **Complete participation** was defined as a pregnant woman attending **pregnancy classes** three times or more and receiving materials on at least seven topics. **Incomplete participation** was defined as a pregnant woman attending pregnancy classes less than three times or receiving material on less than seven topics. Finally, **no participation** was defined as a pregnant woman who did not attend to any pregnancy class at all. The topics included body changes, discomfort during pregnancy, care during pregnancy, childbirth, newborn care, complications, family planning, infectious diseases, local culture related to facts and myths, and birth certificates. All of these are included in Ministry of Health’s guidance of pregnancy class [7, 13].

The covariates used in this study were the mother’s demographic characteristics. Mother’s delivery risk by age was categorized into two categories: high risk, which included mothers whose age increased the likelihood of a complication (under 25 or over 34 years old) and low risk, which included mothers whose age was generally considered safe for pregnancy and childbirth (25–34 years old). Mother’s education was divided into three categories: elementary school or less, junior high school, and senior high school or more. Other control variables were residence site and region. Residence site was categorized as rural or urban; region was categorized as Java-Bali or areas other than Java-Bali. This categorization is based on the proportion of pregnant women in several regions in Indonesia, where the proportion of pregnant women on the islands of Java and Bali is comparable to the number of pregnant women outside of Java and Bali. In addition, we included the number of ANC visits as a covariate in the SBA and FBD usage models. The number of ANC visits was categorized as no visit, 1–3 visits, and 4 or more visits.

#### Statistical analysis

We used descriptive analysis to illustrate the distribution of maternal health service variables and mothers’ demographic characteristics. Logistic and multinomial regression analysis were used to explore the relationship between mothers’ participation on KIH and utilization of adequate ANC, SBA and FBD. We used two different regression models: logistic regression for adequate ANC and SBA utilization, and multinomial logistic regression for FBD utilization. We analysed the data using STATA 12.0. In addition, in all descriptive and estimation analyses we applied the Sirkesnas’s survey weights, which reflect the survey’s complex sample design.

## Results

Table 1 shows the results of our descriptive analysis. 29% of mothers utilized adequate ANC, while 77% of mothers utilized SBA services for their last delivery contact. 44% of the mothers reported giving birth in a health center, while one-third of the mothers used a hospital for their delivery. Most mothers had a middle to higher education level, were in an age range that was less risky for delivery (25–34 years), lived in rural areas, and lived in a region outside Java-Bali. Finally, most claimed to have used adequate ANC at least 4 times. Most of the mothers reported having no participation in the KIH program and only 7% of mothers participated in the complete KIH program.

**Table 1:** Characteristics of the sample

Variables	N (6,790)	%
Utilization of adequate ANC	1989	29.3
Utilization of SBA for last contact	5235	77.1
Utilization FBD in health center	2958	43.6
Utilization FBD in hospital	2180	32.1
Utilization FBD in hospital and health center	1989	29.3
**Mother’s education**		
Finished elementary school or less	2263	33.3
Finished junior high school	1535	22.6
Finished senior high school or more	2992	44.1
**Mother’s delivery risk based on age**		
High risk (14–24 & > = 35)	3115	45.9
Low risk (25–34 years old)	3675	54.1
**Residential site**		
Rural	3709	54.6
Urban	3081	45.4
**Region**		
Other islands	4568	67.3
Java-Bali	2222	32.7
**Number of ANC visits**		
None	266	3.9
1–3 times	2025	29.8
≥ 4 times	4499	66.3
**Level of participation in KIH**		
No participation	5718	84.2
Incomplete participation	610	9.0
Complete participation	462	6.8

Table 2 shows that mothers who participated in the KIH program had higher utilization of adequate ANC, SBA for their last delivery contact, and FBD. Only 27% of mothers who had never participated in the KIH program utilized adequate ANC. Nonetheless, even among mothers who **completed the KIH program,** less than half utilized adequate ANC. A higher proportion of SBA utilization was observed among mothers who completed the KIH program.

**Table 2:** Mother’s utilization of MNH services by level of KIH participation

Factors/ covariates	N	Adequate ANC (%)	SBA (%)	FBD (%)		
				Non-health facility	Health center	Hospital
**Level of participation in KIH**						
No participation	5718	27.10	76.00	25.50	42.30	32.20
Incomplete participation	610	38.40	80.70	20.50	49.50	30.00
Complete participation	462	45.00	86.10	14.50	51.90	33.50

Table 3 summarizes the result of multivariate logistic regression of the possible factors associated with the utilization of adequate ANC and SBA. The results on utilization of adequate ANC show that mothers who completed participation in the KIH program experienced higher odds [OR = 2.19; 95% CI: 1.62 to 2.97; *P* < 0.001] of using adequate ANC than their counterparts who never participated.

**Table 3:** Logistic regression model with Adequate ANC and used SBA

Factors/covariates	N	Adequate ANC		Used SBA for last contact	
		OR	[95% CI]	OR	[95% CI]
***Level of participation in KIH***					
**No (ref)**	5718	1.00		1.00	
**Incomplete**	610	1.64***	[1.17–2.28]	1.76**	[1.14–2.71]
**Complete**	462	2.19***	[1.62–2.97]	2.69***	[1.52–4.76]
***Mother’s education***					
**Finished Elementary school or less (ref)**	2263	1.00		1.00	
**Finished junior high school**	1535	1.36***	[1.08–1.69]	1.91***	[1.41–2.57]
**Finished senior high school or more**	2992	1.61***	[1.29–1.99]	3.11***	[2.27–4.26]
***Mother’s delivery risk based on age***					
**High risk (14–24 & > = 35)**	3115	1.00		1.00	
**Low risk (25–34 years old)**	3675	1.01	[0.82–1.24]	1.19	[0.96–1.47]
***Residential site***					
**Rural (ref)**	3709	1.00		1.00	
**Urban**	3081	0.97	[0.77–1.23]	3.05***	[1.94–4.81]
***Region***					
**Other islands**	4568	1.00		1.00	
**Java-Bali**	2222	1.55***	[1.26–1.90]	2.10***	[1.43–3.09]
***Number of ANC visits***					
**None (ref)**	266			1.00	
**1–4 visits**	2025			6.23***	[4.02–9.68]
**4+ visits**	4499			14.30***	[9.23–22.17]
**Constant**		**0.22**	**[0.18–0.28]**	0.08***	**[0.05–0.14]**

Note: *, **, and *** mark statistical significance at the 10, 5, and 1% levels respectively

Looking at mothers’ characteristics, those who reported having attained a high school education had higher odds [OR = 1.61; 95% CI: 1.29 to 1.99; P < 0.001] of using adequate ANC compared to those with less education. Mothers who lived in the region of Java-Bali had higher odds [OR = 1.55; 95% CI: 1.26 to 1.90; *P* < 0.001] of using adequate ANC compared to those who lived on other islands.

Table 3 also shows that mothers who completed participation in the KIH program had significantly higher odds of using SBA compared to those who never completed it [OR = 2.69; 95% CI: 1.52 to 4.76; *P* < 0.001].

The results on mothers’ other characteristics show that those who reported having attained a high school education had 3.1 higher odds of using SBA [OR = 3.11; 95% CI: 2.27 to 4.26; *P* < 0.001] compared to those who reported having less education. Furthermore, mothers who lived in an urban area had 3.1 higher odds of using SBA compared to those who lived in a rural area [OR = 3.05; 95% CI: 1.94 to 4.81; *P* < 0.001]. Moreover, mothers who lived in the region of Java-Bali had 2.1 times higher odds of using SBA [OR = 2.10; 95% CI: 1.43 to 3.09; P < 0.001] compared to those who lived on other regions. The role of ANC services was also shown to be significantly related to the use of SBA: mothers who received ANC ≥4 visits had higher odds of using SBA compared to those who never had an ANC visit, [OR = 14.30; 95% CI: 9.23 to 22.17; P < 0.001]. In other words, a mother with ANC ≥4 visits had approximately 14 times higher odds of using SBA as their last delivery contact compared to a mother with no ANC visits.

Table 4 shows multinomial logistic regression results, which analyzed the effect of KIH participation on FBD.

**Table 4:** Multinomial logistic regression model with FBD as the outcome

Factors/covariates	n	Health center vs Non-health facility		n	Hospital vs Non-health facility	
		OR	[95% CI]		OR	[95% CI]
***Level of participation in KIH***						
No (ref)	2416	1.00		1842	1.00	
Incomplete	302	1.88***	[1.22–2.91]	183	1.73**	[1.04–2.89]
Complete	240	2.77***	[1.56–4.91]	155	3.19***	[1.74–5.86]
***Mother’s education***						
Finished elementary school or less (ref)	920	1.00		470	1.00	
Finished junior high school	741	1.76***	[1.28–2.40]	422	2.16***	[1.52–3.05]
Finished senior high school or more	1297	2.44***	[1.76–3.38]	1288	4.52***	[3.21–6.37]
***Mother’s delivery risk based on age***						
High risk (14–24 & > = 35)	1322	1.00		971	1.00	
Low risk (25–34 years old)	1636	1.14	[0.91–1.43]	1209	1.13	[0.90–1.43]
***Residential site***						
Rural (ref)	1496	1.00		844	1.00	
Urban	1462	2.76***	[1.74–4.38]	1336	4.13***	[2.66–6.42]
***Region***						
Other islands (ref)	1745	1.00		1364	1.00	
Java-Bali	1213	2.51***	[1.69–3.74]	816	1.87***	[1.27–2.75]
***Number of ANC visits***						
None	29	1.00		16	1.00	
1–4 visits	766	6.87***	[3.95–11.97]	505	6.19***	[3.26–11.75]
4+ visits	2163	16.14***	[9.21–28.29]	1659	14.25***	[7.60–26.74]
Constant		0.04***	[0.02–0.09]		0.02***	[0.01–0.04]

Note: *, **, and *** mark statistical significance at the 10, 5, and 1% levels respectively

The results show that mothers with complete participation in the KIH program had higher odds of using a health center for delivery relative to a non-health facility compared to mothers who had never participated [OR = 2.77; 95% CI: 1.56 to 4.91; *P* < 0.001]. Similarly, mothers who completed the KIH program had 3.2 times higher odds of giving birth in a hospital relative to a non-health facility [OR = 3.19; 95% CI: 1.74 to 5.86; P < 0.001], compared to those who did not participate in KIH.

This study also shows that highly educated mothers had 4.5 times higher odds of giving birth at a hospital relative to a non-health facility compared to those who had less education [OR = 4.52; 95% CI: 3.21 to 6.37; *P* < 0.001]. By comparison, highly educated mothers had only 2.4 times higher odds of giving birth at a health center relative to a non-health facility compared to those who finished elementary school education or less [OR = 2.44; 95% CI: 1.76 to 3.38; *P* < 0.001].

Based on the area of residence, mothers who lived in urban areas had 4.1 times higher odds of giving birth at hospitals relative to non-health facilities compared to those who lived in rural areas [OR = 4.13; 95% CI: 2.66 to 6.42; *P* < 0.001]. Mothers who lived in urban areas had 2.8 times higher odds of giving birth at health centers relative to non-health facilities compared to their rural counterparts [OR = 2.76; 95% CI: 1.74 to 4.38; *P* < 0.001].

Mothers who lived in the Java-Bali region had 1.9 times higher odds of giving birth at a hospital relative to a non-health facility compared to those who lived outside of Java-Bali [OR = 1.87; 95% CI: 1.27 to 2.75; *P* < 0.001]. A similar pattern exists for delivery in health facilities; mothers experienced 2.5 times higher odds of giving birth at health centers relative to non-health facilities compared to those who lived outside of Java-Bali [OR = 2.51; 95% CI: 1.69 to 3.74; *P* < 0.001].

Finally, mothers who received ANC ≥4 visits had 14.2 higher odds of giving birth at a hospital relative to a non-health facility compared to mothers who never received an ANC visit [OR = 14.25; 95% CI: 7.60 to 26.74; P < 0.001], and 16.1 times higher odds of giving birth at a health center [OR = 16.14; 95% CI: 9.21 to 2.29; P < 0.001].

## Discussion

This study assessed the influence of mothers’ participation in the pregnancy class (KIH) program on utilization of MNH services along continuum of care, including adequate ANC, SBA and delivery in health facility. Our results indicate that complete participation in pregnancy classes is associated with higher utilization of adequate ANC, use of SBA as the last contact, and delivery in health center. Thus, effective implementation of the KIH program can support safer pregnancy and delivery.

Our study found that geographical factors also influence the utilization of maternal health services, which may be due to easier access related to distance, number of facilities, and availability of transportation. Our analysis of 2016 Sirkesnas data revealed that the utilization of adequate ANC by mothers was quite low at 29.3%. One possible explanation of low levels of adequate ANC use may be related to some components of the ANC program. For example, case management and examination for sexually transmitted diseases is not conducted systematically among all pregnant women, but rather, only conducted for pregnant women if there is an indication observed beforehand. Similarly, laboratory tests are conducted only when the health facility has the required medical equipment and infrastructure. One study found that the three main reasons for mothers not receiving ANC were: (1) cost/money to pay health services (45.4%), (2) far distance (42.1%), and (3) the mother did not feel a need to check the pregnancy via an ANC visit (34.7%) [21]. Although distance and cost may hinder a mother from accessing ANC, these factors are external and cannot necessarily be controlled by mother. Two approaches may be implemented to improve use of adequate ANC: (1) ensuring the health facilities have high service readiness to deliver ANC services and (2) improving mother’s knowledge on the importance of ANC visits, which can be achieved by improving KIH program implementation.

Our study also shows that the proportion of deliveries assisted by SBA was quite high in Indonesia (77.1%), and most women gave birth in a health facility. Nevertheless, a notable proportion of mothers still used a non-health facility for delivery (24.3%). The possible explanation that such health-seeking patterns persist may be due to the social influences on the mother’s choice of facility for delivery. Research from 2012 indicated that pregnant women generally wished to give birth in health facilities, however, some delivered in non-health facilities because of the influences of parents, husbands, and/or the community [22]. This research also indicates that several other social influences may be important: (1) lack of support from parents or husbands for delivery in health facilities; (2) local cultural norms that consider delivery in health facilities as only for those mothers who experience complications, viewing such births as inconvenient for others in the community. In addition, difficulties in accessing health facilities because of geographical conditions and inadequate transportation may also influence mothers’ decision to deliver in non-health facilities [22]. Other research in Indonesia has shown other reasons mothers choose to give birth with a dukun (a traditional healer), namely, as a result of fewer health workers in the community or less intense interaction with the local community because they live in another village. Cost is also a factor in using a traditional healer, as it costs less to give birth using a traditional birth attendant (called “paraji”) and they are viewed by the community as always ready when needed. Moreover, pregnant women with lower education levels and those from a lower economic level may have limited access to health information, leading to preferences to use a traditional healer [23].

We found that mothers who took KIH classes used adequate ANC at higher rates compared to those who did not take the classes. Based on the 2016 Sirkesnas report, 49.8% of mothers did not know about the KIH program, which may explain the low levels of participation [20]. Pregnancy classes are designed as a means for mothers to improve their knowledge and skills regarding pregnancy, childbirth, and newborns. Before the existence of the KIH program, the problems and complications experienced by pregnant women were handled only on a case-by-case basis through individual consultation. The weaknesses of this process were possibly due to the limited ability of mothers to understand information of ANC, which may be related to low formal education of mothers. However, through the KIH program, mothers’ awareness of the importance of prenatal care increased.

KIH program materials were designed to provide mothers with knowledge of pregnancy problems through discussion of written material on maternal and child health in the form of face-to-face discussions and exchanges of experience. The results of our study confirm that mothers who participate more fully in the KIH program preferred a health facility for giving birth.

In addition, most mothers gave birth with SBA (more than 80%), meaning that they received ANC from health workers in health facilities, so any labor complications could be prevented early on. This result is supported by the analysis from this study showing that mothers involved in KIH had higher odds of obtaining complete ANC services. This result is in line with the results of the study of Juana (2016), which found a meaningful relationship between ANC continuity, maternal education, and parity, with the selection of dukuns as birth attendants. Mothers who did not adhere to ANC, had a low education level, and high parity preferred to use dukuns as the main helpers [24].

Along with increasing mothers’ knowledge about pregnancy, childbirth, and healthy children, the KIH program encourages mothers gain independence decision-making and choosing to give birth using health workers. In addition, they may also provide new insight to their husband and family so that the decision on utilizing the MNH services could be accepted more easily among all family members. Furthermore, full support of family members might increase mothers’ confidence regarding safety, ease, and comfort during labor in health facilities. One study has found lower levels of postpartum depression among mothers involved in KIH compared to mothers not involved in the program [20].

The analysis from this study shows that mothers with complete participation in KIH utilized birth attendants at health facilities, both in the health center and the hospital (51.90 and 33.50% respectively). In other words, our study indicates that the KIH program helped prevent the first delay in the decision to seek care. Thus, it seems that the KIH program as provides many benefits. Even though the observed KIH coverage is low, our analysis shows that increased KIH participation is associated with increased use of MNH services. Our results show us that the difference in the odds between incomplete participation and complete is around 33%, illustrating the significant influence of the KIH program. Thus, the KIH program seems essential to improving the safe utilization of maternal health services. Therefore, the sustainability of the KIH program certainly needs to be balanced with an increase in the quality of facilitators who provide KIH material, especially in terms of effective communication skills and knowledge about the health of pregnancy and childbirth.

One limitation of this research is that it describes conditions only for 2016 or earlier. Despite this limitation, this analysis is the last condition that can be obtained from a national survey specifically relating to ANC and KIH. Also, the Sirkesnas questionnaire had a limited number of questions and lacking some data on several socioeconomic characteristics. Another limitation was that the survey presentation could have resulted in recall bias because of the time elapsed between when the survey was conducted and when women delivered. In addition, there might be issues related to the timing of the ANC. As stated before, the KIH program is introduced to mothers during their second or third trimester of pregnancy. One component of four adequate ANC visits is mothers’ visits during the first, second, and third trimester of pregnancy. We cannot separate the ANC visit in the first trimester to preserve the outcome of ideal ANC as defined by government of Indonesia. We tried to account for this limitation by controlling for factors such as geography, region, and number of ANC visits, however, some omitted variable bias may still exist.

## Conclusion

The KIH program is one of Indonesia’s most important health education programs to direct the utilization of the full MNH continuum of care. Geographical area is an important factor to consider in increasing maternal participation in the KIH program, as easier access may increase the utilization of quality health facilities for maternal and child health. The KIH program is very useful in providing education for pregnant women so they can prepare for safe labor. With this program, mothers increasingly are made aware of the importance of ANC. The results of this study clearly show that increased ANC is related to the percentage of women’s KIH participation, making it imperative to strengthen the existing KIH program. One needed change is to promote the KIH program to the community, both in cities and villages, especially in areas outside of Java-Bali.

We recommend that the KIH program provide education for mothers earlier in their pregnancy, as early as the first trimester. In addition, the information about KIH be disseminated extensively and effectively using various forms of media. The use of mass communication media, such as television, radio, and newspapers, is one way to socialize the program, in addition to community meeting forums (such as “*arisan,*” “*majelis taklim,*” or religious forums) and monthly forums in the villages, as well as social media, used widely by the community. In these ways, information about KIH can be disseminated broadly and quickly. Health program managers should seek to improve the delivery of material in the KIH program to make it more interesting and easier for mothers to understand. Other improvements that could contribute to the success of this program include provision of complete maternal class infrastructure and use of professional human resources to lead KIH sessions.

## Data Availability

With the corresponding text in the availability of data and materials statement: The datasets used and/or analyzed during the current study are available from the corresponding author upon reasonable request. The data that support the findings of this study are available from the Data Management Laboratory, but restrictions apply to the availability, which were used under license for the current study and so are not publicly available. However, data are available from the authors upon reasonable request and with the permission of the Data Management Laboratory, the National Institute of Health Research and Development, Indonesian Health Ministry.

## Abbreviations

ANC: Antenatal care (Indonesian term: Perawatan Kehamilan)
FBD: Facility-based delivery (Indonesian term: Fasilitas Kesehatan untuk melahirkan)
GOI: Government of Indonesia (Indonesian term: Pemerintah Indonesia0
KIH: Pregnancy class (Indonesian term: Kelas Ibu Hamil)
MNH: Maternal and neonatal health (Indonesian term: Kesehatan Ibu dan Anak)
SBA: Skilled birth attendant (Indonesian term: Petugas Kesehatan Penolong Persalinan Terlatih)
SDG: Sustainable Development Goals (Indonesian term: Tujuan Pembangunan Berkelanjutan)
